# Mapping the past, present and future research landscape of paternal effects

**DOI:** 10.1186/s12915-020-00892-3

**Published:** 2020-11-27

**Authors:** Joanna Rutkowska, Malgorzata Lagisz, Russell Bonduriansky, Shinichi Nakagawa

**Affiliations:** 1 Institute of Environmental Sciences, Faculty of Biology, Jagiellonian University, Kraków, Poland; 2 Evolution & Ecology Research Centre, School of Biological, Earth and Environmental Sciences, BEES, The University of New South Wales, Sydney, Australia

**Keywords:** Research weaving, Systematic review, Meta-analysis, Parental effects, Transgenerational effects, Transgenerational plasticity

## Abstract

**Background:**

Although in all sexually reproducing organisms an individual has a mother and a father, non-genetic inheritance has been predominantly studied in mothers. Paternal effects have been far less frequently studied, until recently. In the last 5 years, research on environmentally induced paternal effects has grown rapidly in the number of publications and diversity of topics. Here, we provide an overview of this field using synthesis of evidence (systematic map) and influence (bibliometric analyses).

**Results:**

We find that motivations for studies into paternal effects are diverse. For example, from the ecological and evolutionary perspective, paternal effects are of interest as facilitators of response to environmental change and mediators of extended heredity. Medical researchers track how paternal pre-fertilization exposures to factors, such as diet or trauma, influence offspring health. Toxicologists look at the effects of toxins. We compare how these three research guilds design experiments in relation to objects of their studies: fathers, mothers and offspring. We highlight examples of research gaps, which, in turn, lead to future avenues of research.

**Conclusions:**

The literature on paternal effects is large and disparate. Our study helps in fostering connections between areas of knowledge that develop in parallel, but which could benefit from the lateral transfer of concepts and methods.

**Supplementary Information:**

**Supplementary information** accompanies this paper at 10.1186/s12915-020-00892-3.

## Background

What does ocean acidification have in common with the Dutch famine? They both exert effects that can be non-genetically transmitted from the fathers to their offspring. Publications on such paternal effects (for definitions and nuances, see Table 1) are increasing in number and diversity, with research coming from evolutionary biology [22, 23], medicine [5, 11, 24] and toxicology [25]. Research on paternal effects carried out within those disciplines pursues different goals. For example, evolutionary ecologists seek to understand how paternal effects contribute to heritable variation, how they are influenced by the ambient environment and what role they play in evolution. By contrast, medical and health researchers seek to understand how male health and lifestyle can influence the health of descendants. In each of these disciplines, research is carried out using somewhat different tools and approaches. Cross-fertilization between these disciplines could be very valuable but has been hampered by the use of distinct terminologies and publication outlets.

**Table 1:** Definitions

**a) Disambiguation**	
Paternal effect is a broad term encompassing (i) transgenerational plasticity where the phenotypic change in offspring occurs in response to the paternal environment or phenotype [1], (ii) indirect genetic effects IGEs where alleles expressed in the father affect the development of his offspring [2] and (iii) effects of spontaneous or stochastic variation in non-genetic factors such as epigenetic marks (i.e. variation that is not induced consistently by particular environmental factor). In the current review, we focus on transgenerational plasticity.	
**b) Meaning of paternal effect across the research fields**	
Evolutionary biologists delineate paternal effects most broadly [3]. In this field, a paternal effect reflects the influence of paternal environment or age on offspring traits and can be mediated by paternal care or by factors (such as RNA or proteins) in sperm of seminal fluid. The medical definition usually does not encompass effects transmitted via paternal care [4]. Researchers interested in inheritance of metabolic diseases narrow the definition further into ‘epigenetic programming’ [5] and do not consider age as a part of paternal effects. In terms of the proximate mechanisms, the definitions encompass sperm-borne mechanisms, such as DNA methylation, chromatin alterations and non-coding RNAs [3–6]. In addition, evolutionary perspective is likely to consider mechanisms acting via ejaculate-borne agents, e.g. RNA and proteins, reviewed by [7].	
**c) The term ‘paternal effect’ in other contexts**	
**Identity:** the term is sometimes used to account for paternal identity in statistical models, either in a full-factorial experiment [8] or in studies designed to estimate genetic parameters of sires in animal breeding [9].	
**Genetics:** the term could mean an effect that arises due to the male-specific sex chromosome [10]. The term can denote inheritance of genes through the patriline which exhibits parent-of-origin expression [4], called also ‘epivariation’ [11]. ‘Paternal effect locus’ is a locus whose expression in a male influences the development of his offspring (i.e. an IGE). Recently, it is also referred to as ‘male genetic quality’, related to inbreeding [12].	
**Symbionts/parasites:** although not commonplace, there is evidence that males may transmit symbionts [13] and parasites to their offspring. For instance, males with *Wolbachia* cause embryonic lethality [14]. There is also evidence for paternal mitochondria leakage in animals and humans [15]; these phenomena would be classified as male-specific genetic inheritance, yet to our knowledge have so far not been named ‘paternal effect’.	
**Assisted reproduction:** in assisted reproduction treatment, the term ‘early paternal effect’ refers to failure at the initial stages of the procedure, resulting in zygote malformation, while ‘late paternal effect’ refers to the failure at the stage of implantation [16].	
**d) Interface of epigenetics and genetics**	
Research into paternal effects sheds light on interrelations between different forms of inheritance and their interactions with the environment. First, epigenotype controls the expression of the genotype, while both the genotype and the environment shape the epigenotype [17]. Second, environmentally induced epigenetic processes can promote genetic mutations [18]. Third, factors with mutagenic or cancerogenic effects can also exert epigenetic effects. Exposure to such factors (e.g. smoking) does not allow disentangling the epigenetic effect per se. Finally, classification of effects due to male age is ambiguous. Older males might accumulate effects of lifetime exposure to various environmental [19] and other factors (e.g. exercise). However, older males also have higher numbers of de novo mutations in germline DNA (reviewed, e.g. by [20]) and altered DNA methylation patterns, known as ‘epigenetic clock’ [21], which places age at the interface of genetic and non-genetic factors.	

**Fig. 1:**
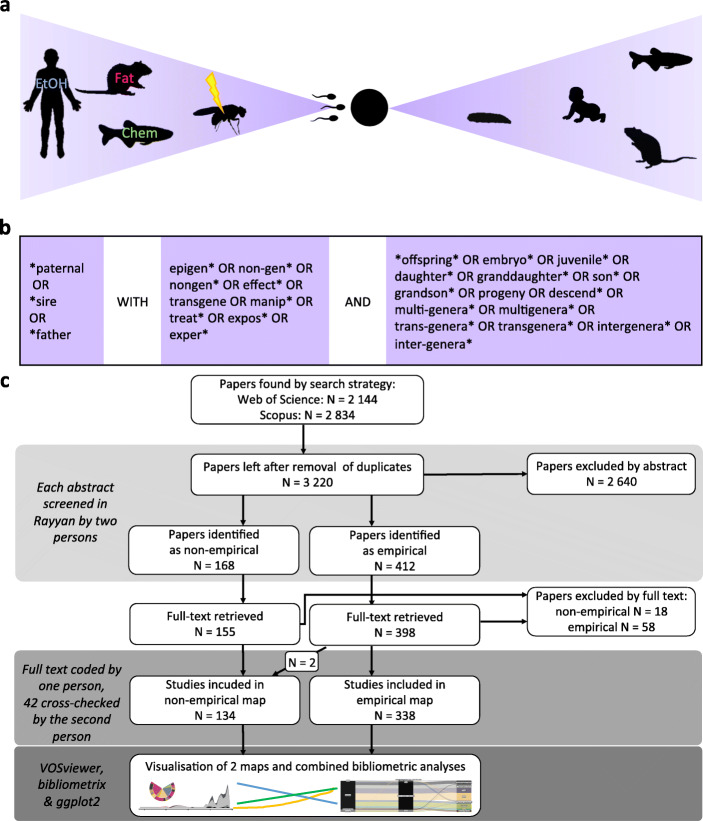
Methods used to create the systematic evidence map of paternal effects research field. **a** The map is based on the published papers on environmentally induced non-genetic paternal germline and semen effects. **b** Keywords used to search the Scopus and Web of Science databases. **c** PRISMA diagram [28] outlining the procedure applied after the literature search

While several thorough and influential reviews of paternal effect research have been published (e.g. [6, 25]), they are focused on a specific type of manipulation eliciting the non-genetic inheritance, or the proximate mechanisms mediating the phenomenon, rarely covering the entire field of paternal effects research. Meta-research (i.e. research on research) could therefore help to identify gaps, biases and clusters in order to facilitate future investigation of paternal effects [26, 27]. Our aim is to construct a systematic and meta-scientific overview of paternal effects research across all relevant fields. Our methodology (Fig. 1) is informed by ‘research weaving’ [29] encompassing synthesis of evidence (systematic map) and influence (bibliometric analyses). A systematic map uses a methodology of literature search similar to that of a systematic review [30, 31]. A systematic map can have a broad scope, allowing for heterogeneity of taxonomic groups and experimental methods, which are usually not consistent across fields. Such a map could include both empirical and non-empirical studies. Adding bibliometric analyses to a systematic map allows assessing networks of ideas and scientists. We use this map to identify research clusters, as well as collaborations that could benefit from cross-disciplinary fertilization of ideas and approaches, particularly between medical and evolutionary-ecological research.

To achieve this goal, we map the past, present and future of the parental effect research. First, we examine temporal and topical trends in the literature and also, via bibliometric analysis, identify three ‘guilds’ (clusters of studies from different research domains). Second, we take a tour of the rather complex experimental landscape, by seeing how the three different guilds design experiments in relation to three family members: fathers, mothers and their children. Third, we highlight three examples of research gaps, which, in turn, lead to future avenues of research. Finally, we offer six considerations for improving future experimental work by integrating insights from our map.

## Results and discussion

### Characterizing temporal, topical and bibliometric patterns

An emerging field of meta-research has recently taught us that, to improve our research practice, we should learn from ‘history’ [29]. To learn the history of a field, our first step is to examine and characterize the trends and patterns in the literature.

#### Temporal trends in paternal effect research

**Fig. 2:**
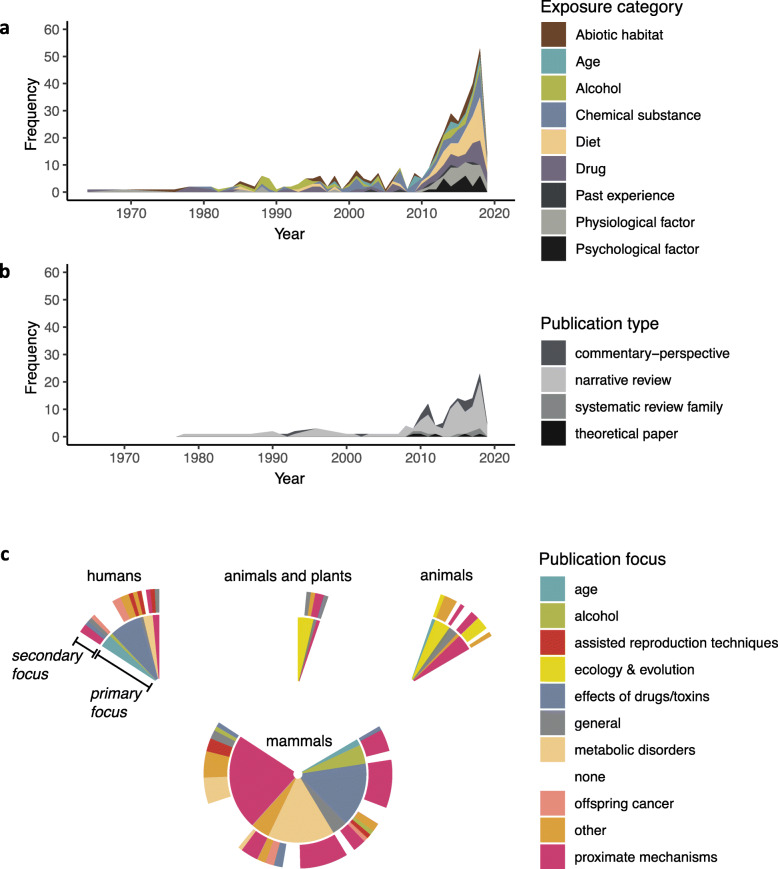
Temporal trends in the map. **a** Timeline of numbers of published empirical papers split by different categories of paternal exposures (the same colour scheme is maintained in Fig. 4). **b** Timeline of numbers of published non-empirical papers split by type of publication. Among non-empirical records, ca. 80% are written as narrative reviews, followed by a smaller number of commentary/perspective works. Very few papers belong to systematic review family, and they are all from a medical cluster (e.g. [32–34]). Theoretical papers, presenting formal models, are even less frequent. The existing ones usually take evolutionary and/or ecological perspective [35–38], with the exception of one focused on the mechanisms of transgenerational inheritance of paternal stress [39]. **c** Primary (inner circle) and secondary (outer circle) topics of non-empirical studies broken down according to major taxonomic groups of considered organisms

Research publications in this field have doubled in number in the last 5 years (Fig. 2). An increasing volume of empirical literature represents the diversity of paternal exposures, with the most pronounced growth in studies investigating trans-generational effects of diet and of psychological factors (Fig. 2a). The growth of empirical evidence is accompanied by the parallel growth of non-empirical papers (Fig. 2b), mostly narrative reviews, with notable scarcity of theoretical papers and systematic reviews (and derivatives [40]). From the secondary literature, we can conclude that the most attention in the field is currently directed towards common health outcomes of paternal exposures: metabolic disorders and detrimental effects of drugs and toxins. Existing reviews often present relatively narrow focus perspective: (1) researchers consider proximate mechanisms of paternal effects to a specific type of exposure, or (2) they associate specific exposure with particular offspring outcomes (Fig. 2c).

#### Three guilds in the paternal effect literature

**Fig. 3:**
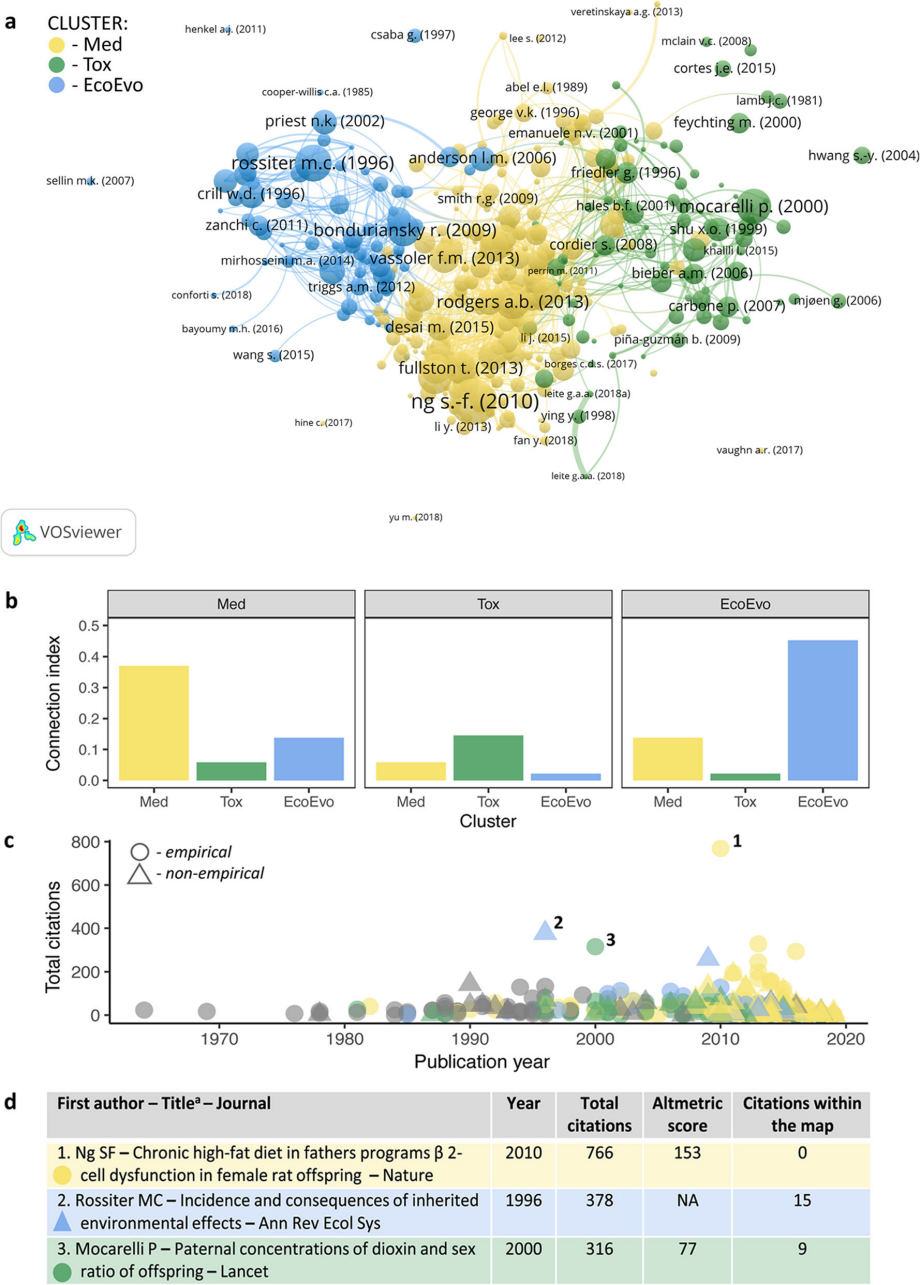
Bibliometric insights into the fragmentation of paternal effect literature. **a** Clustering of paternal effects literature based on bibliometric coupling analysis performed in VOSviewer [41]. We named the clusters based on their dominant research discipline and assigned them different colours, i.e. medical (Med) = yellow, toxicological (Tox) = green and eco-evolutionary (EcoEvo) = blue. **b** Indices of bibliographic connection between papers in the three clusters. **c** Number of citations of papers included in the map. Grey indicates papers not assigned to any cluster. Numbers mark the top cited paper in each cluster. **d** Bibliometric data for the three papers with the highest citation count, one in each cluster; Altmetric Attention Score is a weighted count of all of the online attention

**Fig. 4:**
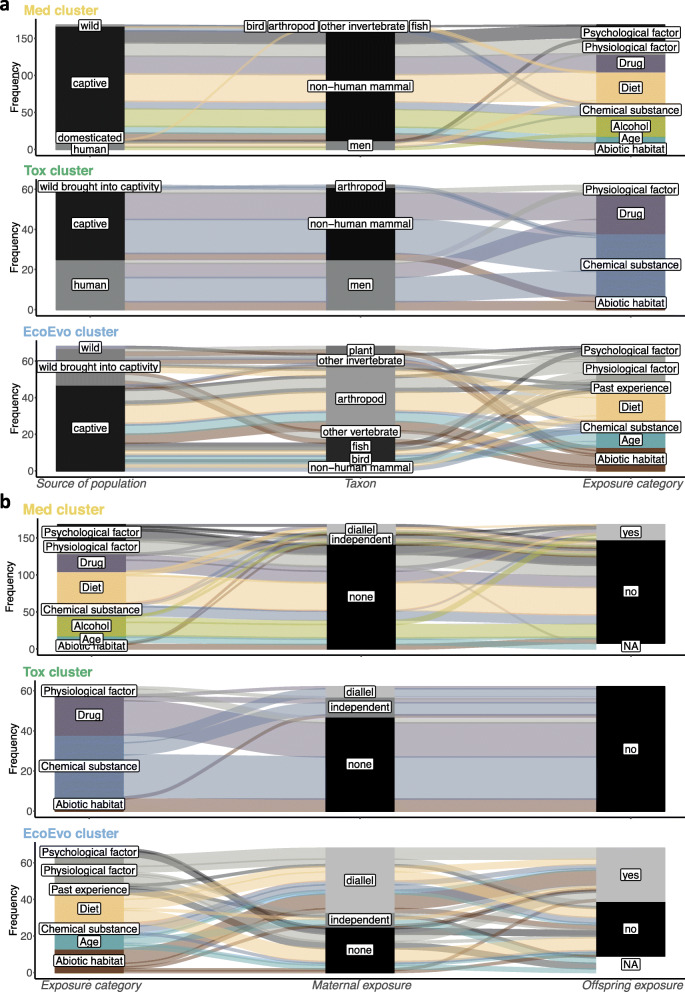
Objects and exposures in paternal effect studies broken down by bibliometric cluster. Plots are based on 302 empirical studies included in the map and assigned to one of the three clusters (Med, Tox and EcoEvo). The size of the panels is proportional to the frequency of studies in a given category. Colour represents the category of experimental exposure (for the legend, see Fig. 2a). **a** Source of studied species, taxonomic group and category of paternal exposure. **b** Category of experimental exposure, information on maternal and offspring exposure to the same factor as the father

As bibliometric clustering algorithm indicated, empirical research in the field of paternal effects has been carried out by three separate guilds, which we call toxicologists, medical scientists and ecology and evolution (eco-evo) researchers (Fig. 3a). Toxicologists maintain the most distinct research guild (Fig. 3b). They typically describe the effects of environmental factors that have negative effects both on the paternal and offspring generations. In this research cluster, there is a substantial share of observational (with a matched control group) studies on humans, while rodents are used as model species in experimental studies (Fig. 4a). We have found that this cluster is the oldest among the three (see Additional File: Fig. S1) and that individual publications are poorly connected even within the cluster (Fig. 3b).

The largest cluster belongs to medical scientists. In principle, we would expect that their studies are similar to those of toxicologists, because paternal exposures are also associated with negative effects on human health. However, a much broader scope of medical research makes their studies markedly different. Rodent studies dominate this cluster with relatively few studies on humans and other taxa (Fig. 4a). Medical scientists differ from toxicologists in their higher propensity for experimental, rather than observational, work. The medical cluster is the youngest and has the highest growth rate (Fig. S1).

The third cluster represents the work of eco-evo researchers. They differ from the other two research groups in also considering paternal effects that might be adaptive for the offspring. Eco-evo researchers have studied various taxonomic groups, including plants, arthropods and other invertebrates, fish, birds and, occasionally, rodents, but they have not studied humans, at least in our map (Fig. 4a). Eco-evo researchers frequently work with organisms in the wild or bring wild animals into captivity (Fig. 4a). The eco-evo cluster has an intermediate temporal distribution and rate of growth of publications (Fig. S1).

#### Influence of and interest in paternal effect research

Analyses of bibliometric influence (expressed as the number of citations per paper, Fig. 3c) show that, in each cluster, there are highly influential studies, both empirical and non-empirical. To exemplify where the attention of the research field is directed, we consider the three papers, ones with the highest number of citations in each cluster (Fig. 3d). Two of those papers present empirical work: one examines the inheritance of metabolic syndrome [42], while the other study examines how dioxin exposure affects offspring sex ratio [43]. The third highly influential paper is a classic review written from the evolutionary perspective [23]. Surprisingly, no articles in our map cite the paper by Ng and colleagues [42], which has the highest total number of citations (mostly by articles belonging to the subject area of “biochemistry, genetics and molecular biology”, as categorized by *Scopus*). The paper [42] demonstrates that paternal high-fat diet consumption leads to the intergenerational transmission of impaired glucose-insulin homeostasis. As such, it would be relevant for researchers studying dietary effects in eco-evo context and those investigating toxicants that alter glucose-insulin homeostasis.

### Landscape of experimental approaches

Experiments on paternal effects pose substantial challenges and complexities. Such experiments should include three parties (fathers, mothers and offspring) in two states (experiment and control), resulting in up to six groups. Notable differences to the most basic experimental design, which involves only one party of subjects divided into experimental and control groups, are twofold. First, researchers apply a treatment to one party of subjects (fathers), but they measure outcomes in different parties (offspring). Second, experiments addressing paternal effects require extra players (mothers), although the researchers usually pay little attention to this extra group. These complications have shaped the current experimental landscape in the paternal effect literature. In this section, we take a tour of this landscape (Fig. 4) through the lens of the three guilds of scientists which we identified in the last section and by following the three ‘main characters’ of the family story: father, mother and offspring.

#### Father

Obviously, fathers (or males) are the heroes of this experimental land. In the description of clusters, we have already uncovered who they are and where they are from. Here, we explore what kinds of challenges (exposures) they experience, and how they experience them.

##### Types of paternal exposure

Eco-evo researchers have a long tradition of studying the paternal effects of diet (Fig. 4a). Also, medical researchers have become increasingly interested in the transgenerational inheritance of the metabolic syndrome due to diet. Naturally, toxicologists have assessed chemical exposure (e.g. pesticides and solvents), mainly in humans, while ecologists measure effects of chemicals (environmental pollutants) on wildlife and non-model species. Somewhat surprisingly, toxicologists have studied the inter/trans-generational effects of medical drugs more than medical scientists. Yet, medical scientists seem to be the only group looking into the effect of paternal alcohol exposure. Both medical researchers and eco-evo researchers have shared their interest in studying (1) physiological exposures via experimental infection and (2) psychological exposures including various social (e.g. isolation or crowding) and physical (e.g. restraint, scent of predator) stressors. Finally, all types of scientists have studied paternal effects in relation to some abiotic aspects, such as water salinity, ambient temperature, electromagnetic field and light exposure.

##### Interactions, dosage and timing of paternal exposures

Most of the time, in a given study, fathers are exposed to only one challenge (94% of all studies). Yet, some researchers in each of the three fields have examined the effects of interaction of different categories of factors acting on the father (for interacting effects between dietary components, see below). For instance, medical researchers have shown that paternal exercise alleviates the negative effect of obesogenic diet in mice [44, 45]. Eco-evo researchers have uncovered complex interactions between paternal age and immune challenge in insects [46].

It is more common to study dose-related responses to a single factor than interactions (approx. 20% of all studies). Toxicologists have always conducted dose-dependency studies [47]. Medical researchers also differentiate dosage in their studies of the effects of paternal alcohol exposure [48, 49]. Similarly, to reveal the effects of paternal age, researchers compare several groups of males of different age [50]. Eco-evo researchers have used gradients of exposures in plant studies [51]. They have also implemented nutritional geometry experiments in animal studies [52, 53], which allow them to reveal non-linear and fine-scale interactive effects of different dietary compounds.

Majority of studies (92% in toxicological, 79% in medical, 66% in eco-evo clusters) manipulate fathers at the adult stage, usually by subjecting them to exposure for one or two cycles of spermatogenesis. Researchers typically use patterns of exposure which mimic what fathers may encounter in real life (e.g. a heat wave [54], different sleep deprivation schedules [55], cocaine intake between weekdays and weekend [56]). Notably, some researchers have manipulated the time passed between exposure and mating. In case of medical drugs, this experimental design allows comparing acute and persistent effects of the exposure [57]. Further, such a design has shown that exposing fathers to chronic stress either at puberty or at adulthood had similar effects on offspring stress axis regulation [58].

#### Mother

As in old fairytales, this heroine has been a rather passive participant in the story. However, she has much to offer and can become a true heroine, as in newer stories. We believe that exciting unexplored possibilities exist when both the hero and the heroine face a challenge (exposure) together.

##### Mate choice and differential allocation

The female can mediate the effects of male experiences in two potential, interrelated ways, one direct and one indirect. Her assessment of male quality can directly affect her prenatal and postnatal maternal investment in offspring [59]. Similarly, yet more indirectly, females could invest in offspring differentially if males induce such response via substances in their ejaculates [3, 4]. Both of these phenomena—via female perception and male substances—are referred to as maternal ‘differential allocation’ and have gained much attention, especially in evolutionary literature [60]. Although female differential allocation is interesting in its own right, to understand the magnitude, function and mechanism of paternal effects, we should limit the opportunity for maternally mediated effects.

Indeed, 77% of researchers across the field have blocked female mate choice by paring up a single male and female (with the exception of human studies). In addition, the researchers predominantly use virgin females (but see [56]). While these two approaches reduce maternal effects due to female perception of male quality, they cannot eliminate them. Eco-evo researchers are most likely to control for differential allocation due to female perception (30%), usually by capitalizing on species with external fertilization [61] or by the means of artificial fertilization in plants, fish and birds [62]. Researchers in medicine control for maternal effects rarely (12%), yet using the greatest variety of methods, including in vitro fertilization [63], embryo transfer [64] and offspring cross-fostering [65]. Toxicologists have rarely dealt with this issue (5%).

Differential allocation induced by male substances is even more challenging to control for, and therefore, only a few have done so to date (e.g. [66]). Nonetheless, researchers could quantify those effects by combining artificial insemination with the use of vasectomized males, which allows assessing the effects of seminal fluid substances. Medical researchers have carried out such studies occasionally [67]. In a similar vein, eco-evo researches have used the so-called telegony approach. Under this approach, a female is mated with two males, both of whom contribute to her offspring phenotypes: the one as a genetic father and the other via semen-mediated effects (only two studies in our collection used this approach [68, 69], see also [70]).

##### Maternal exposure: comparison and synergy

**Fig. 5:**
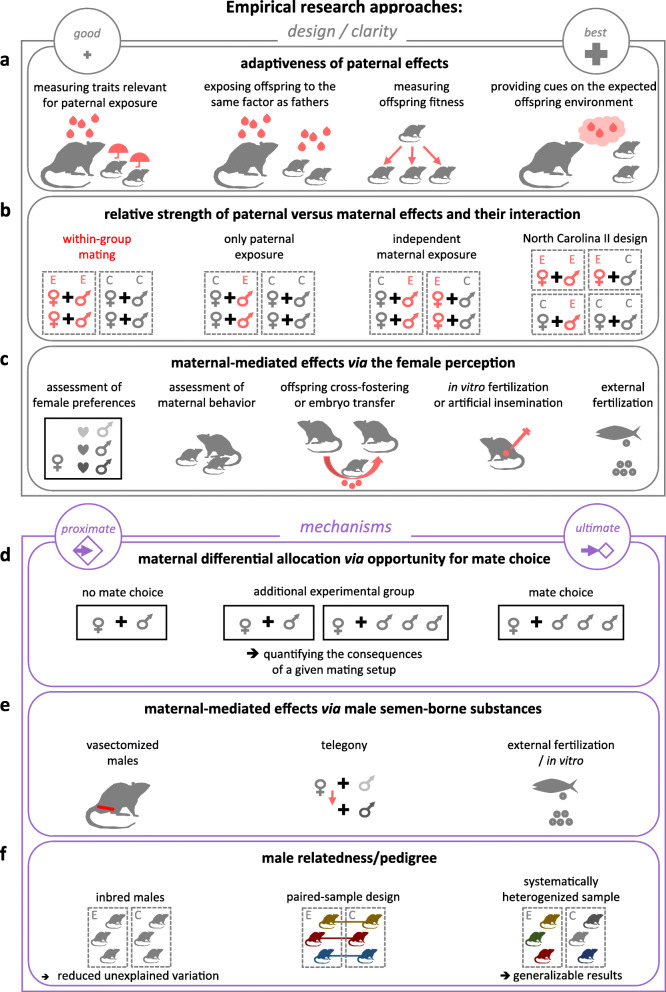
Six useful considerations for paternal effect research. **a** To assess the adaptiveness of paternal effects, measure offspring traits relevant for paternal exposure and, optimally, expose some offspring to the same factor as the father. If possible, study offspring fitness traits. For the best outcomes, include cues that allow prediction of the offspring environment by the fathers. **b** To measure the relative strength of paternal vs. maternal effects, expose female to the same factor as male. Do not mate the parents only within the experimental group (red indicates the design to be avoided). Pair-up exposed parents with control partners to compare maternal and paternal effects. Use North Carolina II design to assess the synergistic effects of both parents. **c** To estimate maternal-mediated effects due to females’ perception, assess female preference for the male and/or maternal behaviour in relation to paternal treatment. Use embryo transfer and offspring cross-fostering. To eliminate effects due to female perception, use in vitro fertilization and artificial insemination. Study species with external fertilization. **d** Allow mate choice, if interested in ultimate aspects of paternal effects. Reduce mate choice, if searching for proximate mechanisms. Add experimental groups to understand the consequences of a particular mating set-up. **e** To reduce maternal-mediated effects due to male semen-borne substances, use vasectomized males, helping identify the proximate mechanism of paternal effects. One could also use telegony approach. To separate female-mediated effects (via male substances and female perception), use species with external fertilization. **f** Use highly related males to reduce unexplained variation and facilitate identification of proximate mechanisms of paternal effects. To obtain robust results, use heterogeneous, randomized sample of males. Using males in a paired-sample design could often be a convenient and powerful option

Researchers can expose mothers to the same challenges as fathers (Fig. 4b). Such a venture opens up possibilities of answering additional questions, but a careful experimental design is warranted. Toxicologists commonly use a design comparing two groups of offspring from biparental exposure vs. non-exposure groups (e.g. [71]). Unfortunately, such a simplistic design precludes assessment of paternal (or maternal) effect alone; accordingly, these studies are not included in our map. Assessing the relative strength of paternal compared to maternal effects is possible when we expose fathers and mothers independently and then pair them up with control individuals (see also Fig. 5f). Sometimes, it is inevitable that exposed partners reproduce only with control (e.g. human medical studies [72, 73];), precluding analyses of the effect of combined exposure.

The most informative is a two-by-two factorial design (also known as North Carolina II). This design enables not only comparing the effect of each parent separately, but also estimating the synergistic (interactive) effect of both [74]. Using the factorial design (55%, Fig. 4b), many of eco-evo researchers have found that the father and mother can have a synergetic effect on offspring (e.g. [75]), but their effects could also cancel each other (e.g. [76]).

#### Offspring

Finally, we turn to the children, who are an essential part of the story, but often neglected. Scientists take many different measurements from the children (offspring) at different times, but they often forget that we have both princes and princesses. Moreover, we find some scientists have also challenged the children, enriching the story plot, while others failed to do so.

##### Measurements: timing, sex-specific and multigenerational effects

Toxicologist, more than others, confine their studies to effects on offspring development; only 30% of their studies track offspring to adulthood. Many medical scientists, in contrast, investigate offspring performance up to adulthood (i.e. 70%, facilitated by the use of relatively fast-maturing lab rodents). Eco-evo researchers also often monitor offspring through development until adulthood (62%), although their monitoring could stop at the juvenile (or larval) stage. Therefore, data on offspring phenotype in the three clusters complement each other, highlighting possibilities of knowledge transfer across the disciplines in this respect.

Researchers who cease the study at early stages of offspring development usually lack information on offspring sex. This, however, only partly explains why over half of the studies of paternal effects do not take into account offspring sex. Although toxicologists have been interested in whether paternal exposure affects sex ratio [77], surprisingly, they are also the least likely to account for offspring sex (34%) in assessing offspring traits. In contrast, medical scientists are the keenest to report effects for the two sexes separately, but also to study only one sex (65% for those two approaches combined). Given the interest in parent-of-origin epigenetic inheritance (Table 1), researchers should routinely examine sex specificity of paternal effects in the offspring [78, 79]. Unfortunately, this is not the case. Instead, the large body of existing literature (63%) has not taken opportunities to detect such sex-specific patterns.

Our map has shown that only ca. 10% of studies examined the transfer of paternal effects to the grand-offspring generation or beyond. Yet, a multigenerational study can provide insights into the nature and persistence of paternal effects. The medical cluster has included such multigenerational studies: the consequences of F0 generation exposure to high-fat diet [80] and heroin addiction [81], in both of which paternal effects were followed up to F3 generation descendants.

##### Offspring exposure: matching, mismatching and beyond

As mentioned earlier, medical scientists and toxicologists have focused on the negative effects of paternal exposures. Thus, it may not be surprising that toxicologists never expose offspring to the same damaging factors (chemicals and drugs, Fig. 4b), although medical scientists have sometimes done so (12%). In contrast, nearly half (47%) of the eco-evo researchers expose offspring to the same factor as fathers. They compare offspring under matched and mismatched conditions to those experienced by their fathers to see if fathers prepare offspring for the same environment via so-called anticipatory paternal effects (sensu [82, 83]). However, to properly investigate anticipatory paternal effects, the experimental design should be based on environmental predictability over the space and time [84]. A proper study should include evidence of the likelihood that the offspring generation will face the same environment as their fathers [84]. In practice, we are aware of no such studies.

### Gaps and opportunities: three examples

We have highlighted what researchers have done so far. Yet, systematic mapping can also elucidate knowledge gaps in the research field [29]. Here, among many potential gaps, we choose to discuss three examples and show how we can turn these gaps into future research opportunities.

#### Oversight over paternal effects in livestock?

Our map, somewhat surprisingly, revealed that paternal effects are neglected in the field of animal breeding (Fig. 4a). In the livestock industry, the choice of sire that produces hundreds of offspring is of paramount importance, and thus, the sire should be of prime quality. Selection schemes of sires usually employ quantitative genetic tools. Thus, much of heritability (due to genetics) is accounted for. However, simultaneous accounting for the epigenome should improve the accuracy of prediction of breeding values. Indeed, among researchers studying farm animals/livestock breeding, there is already an interest in non-genetic paternal inheritance due to sex-specific gene expression patterns [85]; see also Table 1. In terms of environmentally induced paternal effects, it remains unknown what treatment to impose on fathers and which traits to measure in their offspring [17, 86, 87]. Our map could inspire potential research pathways in this field. For example, one of the promising directions would be to explore trans-generational effects related to immunity. So far, research shows that paternal immunization enhances embryonic growth in mice [88], and treating fathers with *Astragalus* polysaccharides increase offspring immunity in the chicken [62]. A recent paper has presented a mathematical model incorporating non-genetic inheritance in livestock breeding [89]. This model could help in designing breeding schemes suitable for investigating non-genetic paternal effects. Last but not least, we could use data from livestock to address the significance of relatedness among males, which is our next topic.

#### Understanding relatedness among fathers for generalisability

Relatedness of studied fathers is approached in a range of ways. They span from use of lab animals without any reference to their inter-relatedness or pedigree (e.g. [90]), use of hybrids of two mouse strains [58], to use of outbred animals [91]. So, what is best? To detect environmentally induced paternal effects, males exposed to the experimental treatments should ideally have counterparts which differ from them as little as possible (Fig. 5f). This design is possible in highly inbred strains. However, findings could be too specific, for example, due to strain-specific reaction norms [92], and thus not transferrable even to other strains of the same species (see also [93]). One solution is to use systematic heterogenization (i.e. controlled and systematic variation of animals and their environment within a single experiment), which improves the representativeness of study individuals [94]. If this is not possible, we recommend assigning full brothers to control and experimental treatment, as in a paired design [95], which results in higher statistical power than in an unpaired design counterpart.

#### In search of paternal bet-hedging

In the face of a stressful and unpredictable environment, mothers should increase variance in offspring traits by employing a so-called bet-hedging strategy [96]. Environmentally stressed fathers should use a similar strategy, as long as the fitness benefits to the male outweigh the costs of investing in such a strategy. Yet, although maternal bet-hedging has been a popular research topic, and the outcomes of the existing studies are mixed [96], we are not aware of any studies examining bet-hedging (via non-genetic effects) by fathers. This gap could be addressed in a number of ways. In terms of empirical studies, the most intuitive approach would be to manipulate the variability of paternal environment and analyse the difference in variance in offspring between treatment groups (i.e. test for heteroscedasticity [97]). Such a study should differentiate between an adaptive male strategy of producing offspring with increased phenotypic variance and a non-adaptive effect of stressful environment on male reproductive physiology. A meta-analytical approach to study paternal bet-hedging is also possible [98], providing that paternal exposures can be unambiguously classified as those that should promote increased or reduced variation in offspring traits. Finally, a recent theoretical model of genomic imprinting [99] predicts reduced variation in offspring phenotype due to paternally (compared to maternally) expressed genes, if males have higher reproductive variance. So far, there are no theoretical models predicting how environmentally induced non-genetic paternal effects affect variation in offspring traits. Thus, such a model is needed.

### Improving paternal effect research for posterity

We have given you a guided tour of our map of the parental effect research through the lens of the three guilds of researchers, three family members and three examples of research gaps. Based on our journey, we offer six considerations for designing future experiments on paternal effects:

- Assessing whether paternal effects benefit offspring health and fitness
- Quantifying paternal, maternal and their interactive effects
- Lessening or eliminating maternally mediated effects via female perception
- Allowing opportunities for mate choice to study maternal differential allocation
- Isolating or eliminating maternally mediated effects via male semen-borne substances
- Considering male relatedness to reduce confounds or enhance generalisability

The first three considerations are useful for singling out paternal effects and clarifying their function, whereas the latter three are concerned with designs suitable for understanding proximate or ultimate mechanisms (Fig. 5). All the considerations provide options depending on researchers’ interest, their study organisms and other logistics. They also provide opportunities for cross-fertilizations of approaches and ideas from the three clusters of scientists. For example, medical researchers often employ sophisticated techniques to elucidate the proximate mechanisms mediating paternal effects [63, 64], and some of these techniques could be utilized by other researchers. Conversely, eco-evo researchers test predictions derived from theory [22, 23] and focus their experiments on ecologically relevant effects. Some of the insights gained from evolutionary and ecological theory could inform the design of medical and toxicological research [4]. Toxicologists typically investigate the effects of a range of treatment levels [47], and such an approach can facilitate the detection of subtle or non-linear effects of the paternal environment on offspring. Such inter-disciplinary links between the three clusters could enhance paternal effect research overall.

## Conclusions

Research into paternal effects is multidisciplinary. However, currently, three relatively insular clusters exist in this research field. We call for more cross-disciplinary collaborations among the three guilds. Further, we note that the importance of paternal effects does not stop at the individual level and that paternally induced changes could propagate into the population and meta-population scales [100]. Altogether, we have much to hope for in the future of the paternal effect research. It will bridge disparate fields of research and will continue to provide useful insights into topics ranging from public health, environmental pollution and climate change to animal science. We can also expect much interest from members of the public by showing that there might be much more than genes to the saying ‘like father, like son’.

## Methods

### Systematic map

The map is based on the published papers on environmentally induced non-genetic paternal germline and semen effects (i.e. when the male had been exposed to some environmental factor before fertilization and the effects were studied in the offspring anytime from the fertilization onwards; Fig. 1a); importantly, it does not include the effects of paternal care, which role is well documented [101–103]. PECO (Population, Exposure, Comparators and Outcomes) statement is available in Additional File: Table S1.

Relevant records were identified via searches carried out in *Scopus* and *Web of Science* databases on 11 April 2019. Sets of keywords are summarized in Fig. 1b, see also Additional File for the exact search string.

The procedure applied after the literature search is presented in a PRISMA diagram [28] (Fig. 3c). In short, we uploaded unique records to Rayyan (https://rayyan.qcri.org/) to perform the initial screening based on the title, abstract and keywords. The screening was done independently by two researchers. We excluded records that did not fulfil all the criteria outlined in the PECO statement. We classified records that fulfilled the inclusion criteria as empirical or non-empirical. We used the Zotero reference manager (https://www.zotero.org/) to retrieve full texts of the designated records. One person coded full texts, with 42 cross-checked by the second person. We uploaded separate datasets of empirical (references [42–58, 61–65, 67–69, 72–77, 80, 81, 83, 88, 90, 91, 104–404]) and non-empirical (references [3–6, 11, 19, 20, 23–25, 32–39, 87, 103, 405–518]) layers into R v.3.6.0 [519] and visualized their content using ggplot2 package [520]. We analysed the combined datasets w using the bibliometrix package [41] and VOSviewer (https://www.vosviewer.com/) [521]. Full details of the methods are provided in the Additional File.

### Bibliometric analyses

We downloaded relevant bibliometric records from* Scopus* database on 16 July 2019. We ran bibliometric coupling analysis in VOSviewer [521] to find clusters in paternal effect literature (Fig. 3a). The unit of analysis was ‘document’ (i.e. each paper). We used a factorial counting method, which equalizes the weight given to each paper, regardless of whether it has been cited, and fractionalization method to visualize the outcome. Clustering resolution was set to 0.8 and minimal cluster size to 60. The resulting number of three clusters was a stable outcome when minimal cluster size parameter was varied between 51 and 79. We named the clusters based on their dominant research discipline, i.e. medical (Med), toxicological (Tox) and eco-evolutionary (EcoEvo).

We calculated the index of bibliographic connection between papers in the three clusters (Fig. 3b) following [522]. The index parameter reflects how many connections are there given the number of all possible connections that could exist between two different clusters and with the cluster itself. The mean connectivity index for our clusters is 0.16 due to low connectivity between clusters and also within clusters themselves. To put this index value into perspective, life-history theory literature, analysed using the same approach, was characterized by a mean index of 0.56 for studies published before 2010 and 0.35 for those published after 2010 [522]. Low connectivity indices may be linked to a rapid increase of volume of available research (although it is not a default relationship), but it may also indicate that literature relevant to a given topic goes unnoticed.

## Supplementary Information

**Additional file 1.** 1. Additional results (Figure S1). Temporal distribution of records belonging to the three clusters. 2. Paternal effects PECO statement regarding empirical papers (Table S1). 3. Search string. 4. Decision trees for initial screening based on abstracts, titles and keywords (Figure S2, Figure S3). 5a. Additional information on selection criteria. 5b. Limitations of the map. 6a. Questionnaire 1, used in full-text coding for the purpose of the map of empirical records. 6b. Questionnaire 2, used in full-text coding for the purpose of the map of non-empirical records. 7. Amendments to the initial protocol. 8a. List of papers excluded based on full text with the reasons – non-empirical layer. 8b. List of papers excluded based on full text with the reasons – empirical layer.

## Data Availability

Data and code are available from https://osf.io/ehy3n/.
