# Supplementary material for: Mapping the past, present and future research landscape of paternal effects
Source: BMC Biol. 2020 Nov 27;18:183. doi: 10.1186/s12915-020-00892-3 (PMC7694421; doi:10.1186/s12915-020-00892-3)
Supplement: Supplementary file 1 — Additional file 1. 1. Additional results (Figure S1). Temporal distribution of records belonging to the three clusters. 2. Paternal effects PECO statement regarding empirical papers (Table S1). 3. Search string. 4. Decision trees for initial screening based on abstracts, titles and keywords (Figure S2, Figure S3). 5a. Additional information on selection criteria. 5b. Limitations of the map. 6a. Questionnaire 1, used in full-text coding for the purpose of the map of empirical records. 6b. Questionnaire 2, used in full-text coding for the purpose of the map of non-empirical records. 7. Amendments to the initial protocol. 8a. List of papers excluded based on full text with the reasons – non-empirical layer. 8b. List of papers excluded based on full text with the reasons – empirical layer. [file 12915_2020_892_MOESM1_ESM.docx]

**Additional file**

**Mapping the past, present and future research landscape of paternal effects**

Joanna Rutkowska^1, 2^*, Malgorzata Lagisz^2^, Russell Bonduriansky^2^, Shinichi Nakagawa^2^

^1^ Institute of Environmental Sciences, Faculty of Biology, Jagiellonian University, Kraków, Poland

^2^ Evolution & Ecology Research Centre, School of Biological, Earth and Environmental Sciences, BEES, The University of New South Wales, Sydney, Australia

emails: joanna.rutkowska@uj.edu.pl, m.lagisz@unsw.edu.au, r.bonduriansky@unsw.edu.au, [s.nakagawa@unsw.edu.au](mailto:s.nakagawa@unsw.edu.au)

* Corresponding author

## Contents

1. Additional results (Figure S1). Temporal distribution of records belonging to the three clusters

2. Paternal effects PECO statement regarding empirical papers (Table S1)

3. Search string

4. Decision trees for initial screening based on abstracts, titles and keywords Figure S2, Figure S3)

5a. Additional information on selection criteria

5b. Limitations of the map

6a. Questionnaire 1, used in full-text coding used in full-text coding for the purpose of the map of empirical records

6b. Questionnaire 2, used in full-text coding used in full-text coding for the purpose of the map of non-empirical records

7. Amendments to the initial protocol

8a. List of papers excluded based on full text with the reasons – non-empirical layer

8b. List of papers excluded based on full text with the reasons –empirical layer

## Additional results

Med Tox Eco-evo


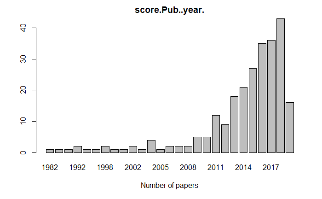


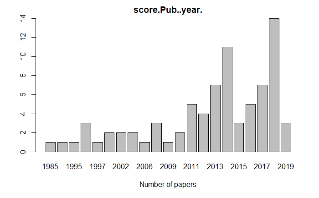

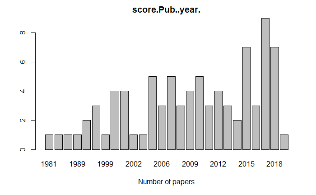


Fig. S1. Temporal distribution (by publication year) of bibliometric records belonging to the three clusters identified in the map.

## 2. Table S1. Paternal effects PECO statement regarding empirical papers.

| **Element** | **Evidence** |
| --- | --- |
| Population | A multicellular species (including humans) with two sexes, in which males are subjected to well-defined manipulation carried out with a proper control group and offspring traits are measured. |
| Exposure | Manipulation of paternal condition/phenotype/state (via extrinsic factors such as temperature, diet, toxins, social environment or intrinsic factors, such as infections, exercise, age and non-heritable life history trait) which occurred before mating/fertilization. In case of species with male pregnancy, paternal exposure should end before fertilization. Excluded are studies in which variation in paternal phenotype has genetic origin (e.g., selection, race, breed, strain, species or mutagenic, carcinogenic agent). Drugs and toxic substances whose effects on germline DNA are uncertain are included. |
| Comparators | Offspring of fathers belonging to the control group in which potential influence of confounding factors was minimalized. |
| Outcomes | Effects which are detected in the offspring from the fertilization onwards, this includes phenotype and fitness components (e.g., gene expression, physiology, morphology, behavior, survival, fecundity). Excluded are studies in which offspring trait results from sex-specific imprinting, parent-of-origin expression, and genes expressed on the sex-specific chromosome or paternal mitochondrial DNA. |

## 3. Search string

Combination of keywords and filters used in Scopus and Web of Science databases on 11th April 2019 (presented in Scopus search format):

TITLE-ABS-KEY ( ( ( *paternal  W/2  epigen* )  OR  ( *paternal  W/2  non-gen* )  OR ( *paternal  W/2  nongen* )  OR  ( *paternal  W/2  effect* )  OR  ( *parental  W/2  transgene )  OR  ( *paternal  W/2  manip* )  OR  ( *paternal  W/2  treat* )  OR  ( *paternal  W/2  expos* )  OR  ( *paternal  W/2  exper* )

OR  ( *sire  W/2  epigen* )  OR  ( *sire  W/2  non-gen* ) OR ( *sire  W/2  nongen* )  OR  ( *sire  W/2  effect* )  OR  ( *sire  W/2  transgene* )  OR  ( *sire  W/2  manip* )  OR  ( *sire  W/2  treat* )  OR  ( *sire  W/2  expos* )  OR  ( *sire  W/2  exper* )

OR ( *father*  W/2  epigen* )  OR  ( *father*  W/2  non-gen* )  OR ( *father*  W/2  nongen* )  OR  ( *father*  W/2  effect* )  OR  ( *father*  W/2  transgene* )  OR  ( *father*  W/2  manip* )  OR  ( *father*  W/2  treat* )  OR  ( *father*  W/2  expos* )  OR  ( *father*  W/2  exper* ) )

AND  ( *offspring*  OR  embryo*  OR  juvenile*  OR  daughter*  OR  granddaughter*  OR  son*

OR grandson*  OR  progeny  OR  descend*  OR  multi-genera*  OR  multigenera*  OR  trans-genera*  OR  transgenera* OR *intergenera** OR *inter-genera**) )

## 4. Decision trees


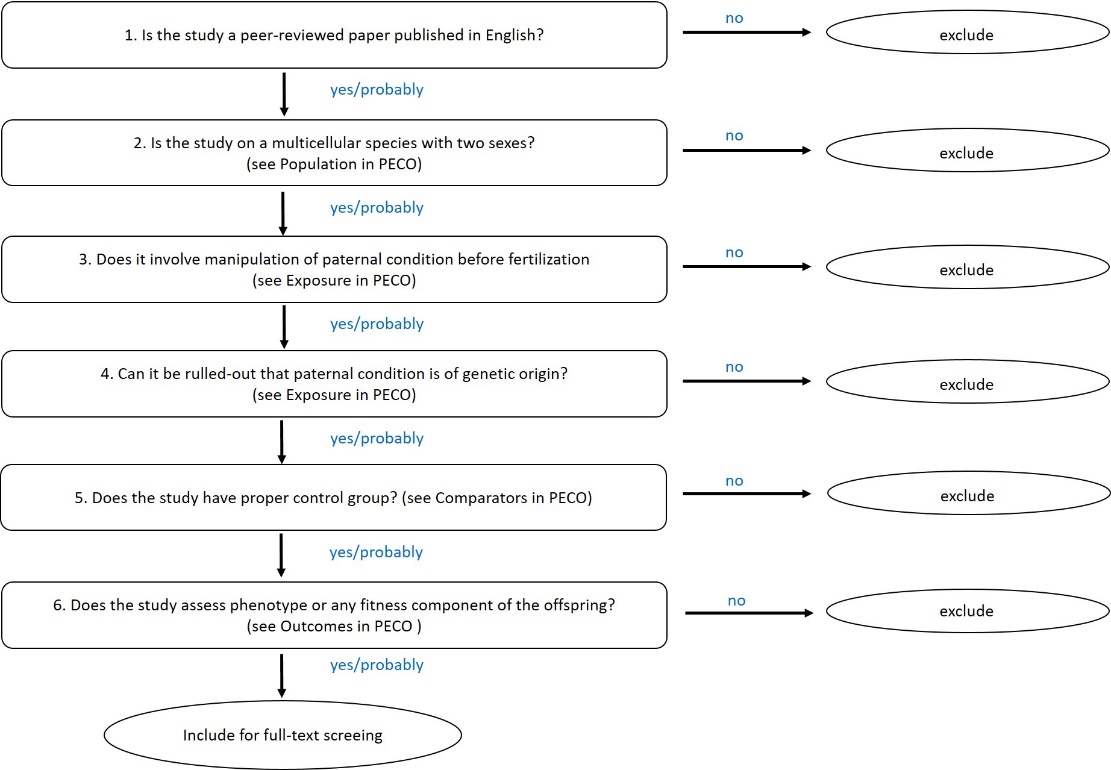


Figure S2. Decision tree for Stage 1 screening (based on title, abstract and keywords) records of empirical studies.


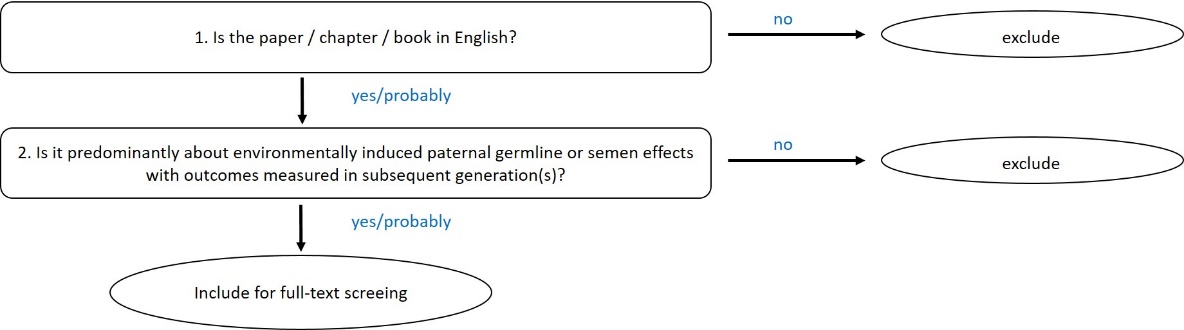


Figure S3. Decision tree for Stage 1 screening (based on title, abstract and keywords ) records of non-empirical studies.

## 5a. Additional information on selection criteria

To date, most reviews divide types of factors that underlay paternal effects into categories of dietary exposure, drugs/toxins and age. We aimed at providing a more detailed list of exposure categories, including, e.g., features of abiotic habitat, factors which may result from fathers’ past experience of intrinsic character or of extrinsic character. In order to allow identification of studies with the possibility of sperm competition, and in which experimental approach mimics or disrupts the natural mating system of the species, we noted the social mating system of the species and the way in which mating was carried out in a given study. We also marked the stage at which father was exposed to the experimental factor, because this might be relevant for the inference of the proximate mechanism mediating paternal effect and for assessing the persistence of the change in paternal condition. In some taxa (especially mammals) it has been demonstrated that epigenetic germline inheritance is carried to the grand-offspring generation (ref. 85 in the main document). This is most often true for the maternal effects, but some studies report it also for the exposed males (ref. 3 in the main document). Thus, the map aims to identify studies that followed consequences of paternal effects beyond F1 generation.

It is rarely possible to disentangle the relative contribution of paternal effects acting directly from those acting via maternal effects or in interaction with them (review by ref. 32 and 77, but see refer. 56 in the main document). We assessed the potential for such disticnition by recording whether studied species have external or internal fertilization and whether in-vivo versus in-vitro fertilization was used. Finally, we outlined other ways to control (or partly control) for maternal effects and other indirect genetic effects, such as offspring cross-fostering.

Paternal care is most likely the first recognized paternal effect, and its role is relatively well documented (ref. 91, 92 in the main document), including studies which show that deprivation of paternal care affects offspring phenotypes (behaviour and neural development; ref. 93 in the main document).

The empirical layer of the map was meant to cover only experimental studies published as peer-reviewed papers. Fathers in the control group should differ from the fathers of the treatment group only in the exposure to a specific environmental factor of interest, so that influence of other confounding factors is minimized. For instance, in case of animal studies, studies that look at the age effect of fathers should be carried out in the laboratory, so that animals only differ in their age but not in other traits. In case of human studies, randomized or quasi-randomized trials are required. For example, studies that look at father’s exposure to a toxic substance should have control group with matched race, age, economic status, etc. We made a priori decision not to include indirect genetic effects. Those effects are mediated by a change in paternal care or a change in the seminal fluid, etc., and will not be included if not induced by paternal environment.

Non-empirical layer was designed to include secondary type of publications covering topics outlined above for the empirical papers. This was verified using a decision tree for non-empirical studies (Figure S3). Narrative reviews were defined as publications starting from few pages long and could be an opinion piece that referenced several studies. The category of commentary-perspective records was defined as short paper describing recent findings of the other authors. Papers that included mathematical models were categorised as theoretical.

More detailed information on exclusions:

1. Environmental factors which are known to cause genetic mutations (such as radiation and mutagenic agents), unless it was possible to disentangle potential epigenetic effects of such factors from the effects of mutations.
2. Effects caused by selection, such as race, breed, strain, species, all of which have significant genetic component. Life-history strategies which are known to have genetic origin e.g. heritable migratory tactics in salmonid fishes were also excluded.
3. Effects of sex-specific imprinting, parent-of-origin expression (called also “epivariation”, ref. 5 in the main document), and genes expressed on the male-specific sex chromosome. These are sometimes referred to as “paternal effects”, but they do not fit into the definition of being under the influence of paternal condition, unless the pattern of inheritance is affected by environmental exposure of the father (ref. 16 in the main document).
4. One of the approaches to study the proximate mechanism of paternal non-genetic inheritance, especially mediated by seminal fluids is in situations in which offspring are affected by the condition of a male who is not their father (but is, for example, female’s past mate; ref. 17 and 58 in the main document). Those studies were identified as one of the categories among ways of assessing the proximate mechanism of paternal effects.

## 5b. Limitations of the map

By the nature of systematic map, we aimed at the most objective approach of presenting the field. However, the choice of search keywords, categorization of the records and tools used during bibliometric analyses are subjectively driven by the curiosity and experience of the authors. We acknowledge that we could have missed some relevant records. For instance, additional searchers carried out at the stage of manuscript revision, revealed that adding phrases ( *paternal  W/2  sperm* ) OR (paternal  W/2  transmission* ) OR ( *paternal  W/2  program* ), would result in ca. 8% more studies included in the map. Importantly, the additional records are not qualitatively different from the records in the original dataset.

Our findings could be biased if there were some underlying features in the dataset that we did not accounted for in our categorization. For one, we assumed that the papers included in the map are representative for the research effort in the field. However, we did not search for the unpublished studies. One of the predominating features of meta-analytical methods is incorporation of sample size on which the evidence is based (ref. 505 in the main document). In our map, individual papers are treated equally irrespective on the sample on which there are based. An evidence map can also consider critical appraisal of the studies from a perspective of reporting standards, a nice example of such approach is ref. 509 in the main document) referenced in the main document in the non-empirical layer of our map. Type of biases mentioned above (publication bias towards statistically significant findings, inequality of sample sizes and quality of studies) should be considered in the narrower field, when it is more feasible to compare individual studies. Ultimately, a meta-analysis can be used to quantify outcomes of the selected studies.

From technical perspective, the map is limited to records covered in the two publication databases that we have used. The choice of databases was dictated by the possibility of using the similar string of search keywords, which would not be possible, for instance in the PubMed. (MeSH terms-based, “Paternal Inheritance” query, carried out in the PubMed database, revealed only additional 1% studies compared to the original database). Thus, some older literature is probably missing. Also, as Scopus does not support extraction of books’ citations, books or book chapters which are included in the non-empirical layer of the map are not covered by the bibliometric analyses (ref. 411 in the main document). However, as in case of statistical analysis, we do not need all population of interest, but we require a representative and unbiased sample from the population (which we emphasized in ref. 9 in the main document). This, we believe, is the case for our sample of literature on paternal effects.

Finally, we would like to stress that the literature on paternal effects is rapidly expanding, with new studies being published at an accelerating rate. Nonetheless, our review’s main messages are likely to remain true for many years to come.

## 6a. Questionnaire 1, used in full-text coding for the purpose of the map of empirical records

### Instructions:

(i) Obligatory questions are marked with “*”.

(ii) Some papers might fall into more than one category. Such cases will be coded in the questionnaire using multiply choice questions, e.g., paternal condition can be simultaneously manipulated using ambient temperature and diet.

(iii) Open-filed entries for a given paper should be separated by semicolon (*Mus musculus*; *Rattus norvegicus*).

(iv) Some questions have options “other”. We reserve the possibility that if over 20% of response for a given question fall into that category, we will try to introduce new, more specific response option.

### Questions:

* Automatic/semiautomatic extraction of year, source (usually journal name), author names, country (of the corresponding author), author keywords, and number of citations

1. Does the study meet criteria of the map? If NOT, give the reasons for exclusion and submit the response without answering other questions
2. * Taxon (choose from): plant / insect / fish / amphibian / bird / non-human mammal / human / other
3. * Scientific name: insert
4. Social mating system of the species: monogamy / polygamy / promiscuity / sex-role reversal system
5. * Mating carried out in the current study: with mate-choice / without mate choice / unclear
6. * Source of population: wild / wild brought into captivity / captive (for more than one generation) / domesticated / human / NA
7. * Stage at which the father was exposed to the experimental factor? in utero / as juvenile / as adult
8. * Type of factor describing/affecting paternal condition:

- Abiotic features of the habitat, e.g., temperature, salinity, environmental enrichment
- Nutrients which are naturally available for a given species, e.g., type of food or its quantity, amount of specific nutrients, such as protein, fat, carbohydrates, micronutrients, vitamins
- Alcohol, drugs, toxins, endocrine disruptors and other substances which are not encountered by a given species in its natural conditions
- Experimentally induced intrinsic factors affecting physiology of an organism, e.g. hormone levels, infections, exercise, sleep
- Experimentally induced psychological factors, such as social environment
- Experimentally induced individual past experience, e.g., previous mating experience, as well as life-history strategy (if for a given species it is not genetically determined)
- Age
- Other

1. * Specific factor describing/affecting paternal condition (e.g., water salinity, fat content age): insert
2. * Does the study attempt to assess the proximate mechanism of paternal effect? does not assess / correlative assessment (e.g., measurements of sperm traits) / assessment using telegony / assessment using male pregnancy / experimental alteration of a specific sperm/semen trait / other
3. * Does the study attempt to exclude/control the maternal effects or other indirect genetic effects? no / yes – maternal effects/ yes – indirect genetic effects / unclear
4. If so, how does study control for maternal effects or other indirect genetic effects? in-vitro fertilization / natural external fertilization / artificial insemination / cross-fostering / other
5. * What is the latest stage at which offspring were measured? embryos / just born-hatched / larvae / juveniles / adults
6. How does the study assess phenotype or fitness component of the offspring (indicate for each stage of the offspring development, (survival should be assigned to the stage at the end of which it was assessed)? gene expression / physiology / morphology / behavior / survival / fecundity / other
7. * Traits measured in the offspring (e.g., level of antibodies, growth rate, cognition, size of sexual ornaments, mating success): insert
8. * Does the study assess grand-offspring phenotype or fitness component? phenotype / fitness (survival or fecundity) / phenotype & fitness (survival or fecundity)
9. If so, what was the latest stage at which the grand-offspring were studied? embryos / just born-hatched / juveniles / adults
10. * Does the study report sex-specific effects in the progeny? no / yes / one sex measured only

## 6b. Questionnaire 2, used in full-text coding for the purpose of the map of non-empirical records

### Instructions:

Obligatory questions are marked with “*”.

### Questions:

* Automatic/semiautomatic extraction of year, source (usually journal name), author names, country (of the corresponding author), author keywords, and number of citations

1. Does the study meet criteria of the map? If NOT, give the reasons for exclusion and submit the response without answering other questions
2. * What publication form does the record have? book / chapter / peer-reviewed article / other
3. * What type of paper is it or claims to be?: commentary-perspective (e.g. short paper describing recent findings of the other authors)/narrative review (stating from few pages long, can be an opinion piece that references several studies)/systematic review family/theoretical paper (including mathematical models)
4. * What is its taxonomic scope? (e.g., choose “animals” if the paper refers to some non-mammalian studies): humans / mammals / animals, / animals and plants
5. * What is its primary focus?: metabolic disorders / effects of drugs or toxins / age / proximate mechanism of paternal effects / assisted reproduction techniques / agriculture & animal breeding / ecology & evolution / other
6. If the last answer was “other”, insert short response here: ….
7. What is its secondary focus?: metabolic disorders / effects of drugs or toxins / age / proximate mechanism of paternal effects / assisted reproduction techniques / agriculture & animal breeding / ecology & evolution / other / none
8. If the last answer was “other”, insert short response here: ….

## 7. Amendments to the initial protocol

1. After screening of abstracts the following change was introduced in the coding of full texts:

| Was: | Changed into: |
| --- | --- |
| * Type of factor describing/affecting paternal condition:   - Alcohol, drugs, toxins, endocrine disruptors and other substances which are not encountered by a given species in its natural conditions | * Type of factor describing/affecting paternal condition:   - alcohol - drugs - chemical substances, incl. e.g. pesticides, solvents and other substances which are not encountered by a given species in its natural conditions |
| -- | 19. Whether and how does the study exposed mothers to the same factors as fathers? |
| -- | 20. Other comments |

b) After coding full texts of non-empirical records, the following changes were introduced in the categories of primary and secondary focus:

| Was: | Changed into: |
| --- | --- |
| effects of drugs | effects of drugs/toxins |
| subset of “drugs” | alcohol |
| subset of “other” | general |
| subset of “other” | offspring cancer |
| agriculture & animal breeding | merged with “other” |

1. After coding full texts of empirical records, the following changes were introduced

| Was: | Changed into: |
| --- | --- |
| 2. Taxon:  amphibian  other  insects | 2. Taxon:  other vertebrates  other invertebrates  arthropods |
| 4. Social mating system of the species separate categories polygamy / promiscuity | single category polygamy – promiscuity |
| -- | 21. Where offspring exposed to the same factor as their fathers? |

## 8a. List of papers excluded based on full text with the reasons – non-empirical part

|  | Reference | Reason for exclusion |
| --- | --- | --- |
| 1 | Bellinger, D. C. 2005. Teratogen update: Lead and pregnancy. Birth Defects Research Part A - Clinical and Molecular Teratology 73:409-420. | mostly maternal exposures |
| 2 | Brown, N. A. 1985. Birth defects: Are offspring at risk from their father's exposure to toxins? Nature 316:110. | exposure to the mutagenic drug |
| 3 | Crean, A. J., M. I. Adler, and R. Bonduriansky. 2016. Seminal Fluid and Mate Choice: New Predictions. Trends in Ecology and Evolution 31:253-255. | not on paternal environmental exposure prior to conception |
| 4 | Fitch, K. R., G. K. Yasuda, K. N. Owens, and B. T. Wakimoto. 1997. Paternal Effects in Drosophila: Implications for Mechanisms of Early Development. Pages 1-34. | not on paternal environmental exposure prior to conception |
| 5 | Giudice, L. C. 2016. Environmental toxicants: hidden players on the reproductive stage. Fertility and Sterility 106:791-794. | no much about paternal germline exposure |
| 6 | Heynick, L. N., and J. H. Merritt. 2003. Radiofrequency Fields and Teratogenesis. Bioelectromagnetics 24:S174-S186. | not on paternal environmental exposure prior to conception |
| 7 | James, W. H. 2000. Exposure to chemicals, offspring sex ratios, and their relevance to teratology. Teratology 62:75-76. | not predominantly about paternal germline effects |
| 8 | Miller, D., M. Brinkworth, and D. Iles. 2010. Paternal DNA packaging in spermatozoa: More than the sum of its parts? DNA, histones, protamines and epigenetics. Reproduction 139:287-301. | not on paternal environmental exposure prior to conception |
| 9 | Piersma, A. H., E. Rorije, M. E. W. Beekhuijzen, R. Cooper, D. J. Dix, B. Heinrich-Hirsch, M. T. Martin, E. Mendez, A. Muller, M. Paparella, D. Ramsingh, E. Reaves, P. Ridgway, E. Schenk, L. Stachiw, B. Ulbrich, and B. C. Hakkert. 2011. Combined retrospective analysis of 498 rat multi-generation reproductive toxicity studies: On the impact of parameters related to F1 mating and F2 offspring. Reproductive Toxicology 31:392-401. | no separation between paternal and maternal exposure |
| 10 | Rodakis, G. C. 2013. Paternal Inheritance. Pages 238-239. | not on paternal environmental exposure prior to conception |
| 11 | Russell, S. D., X. P. Gou, X. P. Wei, and T. Yuan. 2010. Male gamete biology in flowering plants. Biochemical Society Transactions 38:598-603. | not on paternal environmental exposure prior to conception |
| 12 | Schrader, M., M. Müller, B. Straub, and K. Miller. 2001. The impact of chemotherapy on male fertility: A survey of the biologic basis and clinical aspects. Reproductive Toxicology 15:611-617. | not predominantly on subsequent generation |
| 13 | Scialli, A. R., G. Bailey, B. K. Beyer, I. B. Bøgh, W. J. Breslin, C. L. Chen, A. M. DeLise, J. Y. Hui, G. J. Moffat, J. Stewart, and K. E. Thompson. 2016. Reprint of "Potential seminal transport of pharmaceuticals to the conceptus". Reproductive Toxicology 59:22-30. | not predominantly of the effects on subsequent generation (applies also to the version from 2015) |
| 14 | Simpson, S. J., A. R. McCaffery, and B. F. HÄgele. 1999. A behavioural analysis of phase change in the desert locust. Biological Reviews 74:461-480. | only one short paragraph on paternal effects |
| 15 | Simsek, M., C. B. Lambalk, J. A. Wilschut, C. J. J. Mulder, and N. K. H. De Boer. 2018. The associations of thiopurines with male fertility and paternally exposed offspring: A systematic review and meta-analysis. Human Reproduction Update 24:192-206. | mutagenic effects of the reviewed drug |
| 16 | Sinclair, K. D., A. Karamitri, D. S. Gardner, M. C. Lucy, J. L. Pate, M. F. Smith, and T. E. Spencer. 2010. Dietary regulation of developmental programming in ruminants: epigenetic modifications in the germline. Pages 59-72. | hardly any information on paternal effects |
| 17 | Smith, G. D., S. Leary, A. Ness, and D. A. Lawlor. 2009. Challenges and novel approaches in the epidemiological study of early life influences on later disease. Pages 1-14. | not on paternal environmental exposure prior to conception |
| 18 | Steger, K., M. C. O. Cavalcanti, and H. C. Schuppe. 2011. Prognostic markers for competent human spermatozoa: Fertilizing capacity and contribution to the embryo. International Journal of Andrology 34:513-527. | not on paternal environmental exposure prior to conception |

## 8b. List of papers excluded based on full text with the reasons –empirical part

|  | Reference | Reason for exclusion |
| --- | --- | --- |
| 1 | Besson, A. A., R. Guerreiro, J. Bellenger, K. Ragot, B. Faivre, and G. Sorci. 2014. Parental experience of a risky environment leads to improved offspring growth rate. Journal of Experimental Biology 217:2734-2739. | effects of paternal exposure cannot be separated from maternal one |
| 2 | Bolhuis, K., S. A. Kushner, S. Yalniz, M. H. J. Hillegers, V. W. V. Jaddoe, H. Tiemeier, and H. El Marroun. 2018. Maternal and paternal cannabis use during pregnancy and the risk of psychotic-like experiences in the offspring. Schizophrenia Research 202:322-327. | effects of paternal exposure to cannabis before conception were not reported |
| 3 | Cole, E. L., I. Ilies, and R. B. Rosengaus. Competing Physiological Demands During Incipient Colony Foundation in a Social Insect: Consequences of Pathogenic Stress. Frontiers in Ecology and Evolution 6. | no data on offspring phenotype (egg volume cannot be considered as such) |
| 4 | Crijns, I., J. Bos, M. Knol, S. Straus, and L. De Jong-Van Den Berg. 2012. Paternal drug use: Before and during pregnancy. Expert Opinion on Drug Safety 11:513-518. | no offspring performance traits were assessed |
| 5 | Dong, X., Z. Zhang, S. Meng, C. Pan, M. Yang, X. Wu, L. Yang, and H. Xu. 2018. Parental exposure to bisphenol A and its analogs influences zebrafish offspring immunity. Science of the Total Environment 610:291-297. | effects of paternal exposure cannot be separated from maternal ones |
| 6 | Dunn, G. A., and T. L. Bale. 2011. Maternal high-fat diet effects on third-generation female body size via the paternal lineage. Endocrinology 152:2228-2236. | experimental design does not allow to compare in utero exposed and unexposed males |
| 7 | El Marroun, H., K. Bolhuis, I. H. A. Franken, V. W. V. Jaddoe, M. H. Hillegers, B. B. Lahey, and H. Tiemeier. 2019. Preconception and prenatal cannabis use and the risk of behavioural and emotional problems in the offspring; A multi-informant prospective longitudinal study. International Journal of Epidemiology 48:287-296. | no proper control group |
| 8 | Emlen, D. J. 1994. Environmental control of horn length dimorphism in the beetle Onthophagus acuminatus (Coleoptera: Scarabaeidae). Proceedings of the Royal Society B: Biological Sciences 256:131-136. | male morph was not induced experimentally |
| 9 | Falk, M. C., X. H. Zheng, D. L. Chen, Y. Jiang, Z. S. Liu, and K. D. Lewis. Developmental and reproductive toxicological evaluation of arachidonic acid (ARA)-Rich oil and docosahexaenoic acid (DHA)-Rich oil. Food and Chemical Toxicology 103:270-278. | no data regarding effects of paternal exposure on the offspring are presented |
| 10 | Farag, A. T., N. F. Goda, A. H. Mansee, and N. A. Shaaban. 2006. Effects of permethrin given before mating on the behavior of F1-generation in mice. NeuroToxicology 27:421-428. | effects of paternal exposure cannot be separated from maternal ones |
| 11 | Farag, A. T., N. F. Goda, N. A. Shaaban, and A. H. Mansee. 2007. Effects of oral exposure of synthetic pyrethroid, cypermethrin on the behavior of F1-progeny in mice. Reproductive Toxicology 23:560-567. | effects of paternal exposure cannot be separated from maternal ones |
| 12 | Foldi, C. J., D. W. Eyles, J. J. McGrath, and T. H. J. Burne. 2011. The effects of breeding protocol in C57BL/6J mice on adult offspring behaviour. PLoS ONE 6. | the study does not clearly manipulate paternal condition prior to conception |
| 13 | Forest, A. R., M. G. E. Dender, T. E. Pitcher, and C. A. D. Semeniuk. 2017. The effects of paternal reproductive tactic and rearing environment on juvenile variation in growth as mediated through aggression and foraging behaviours of Chinook salmon (Oncorhynchus tshawytscha). Ethology 123:329-341. | no description of whether male phenotype (reproductive tactic) was induced experimentally |
| 14 | Ganiger, S., H. N. Malleshappa, H. Krishnappa, G. Rajashekhar, V. Ramakrishna Rao, and F. Sullivan. 2007. A two generation reproductive toxicity study with curcumin, turmeric yellow, in Wistar rats. Food and Chemical Toxicology 45:64-69. | effects of paternal exposure cannot be separated from maternal ones |
| 15 | Ghanayem, B. I., R. Bai, G. E. Kissling, G. Travlos, and U. Hoffler. 2010. Diet-induced obesity in male mice is associated with reduced fertility and potentiation of acrylamide-induced reproductive toxicity. Biology of Reproduction 82:96-104. | no offspring traits (except their number) were measured |
| 16 | Jaffe, B., D. Shye, S. Harlap, M. Baras, E. Belmaker, L. Gordon, S. Magidor, and J. Fortneys. 1990. Health, growth and sexual development of teenagers exposed in utero to medroxyprogesterone acetate. Paediatric and Perinatal Epidemiology 4:184-195. | no effects on the offspring were measured |
| 17 | Kendig, E. L., D. R. Buesing, S. M. Christie, C. J. Cookman, R. B. Gear, E. R. Hugo, S. N. Kasper, J. A. Kendziorski, K. R. Ungi, K. Williams, and S. M. Belcher. 2012. Estrogen-like disruptive effects of dietary exposure to bisphenol A or 17α-ethinyl estradiol in CD1 mice. International Journal of Toxicology 31:537-550. | effects of paternal exposure cannot be separated from maternal ones |
| 18 | Kobayashi, T., M. Takano, K. Kaneko, and M. Onoue. 2014. A one-generation reproduction toxicity study in rats treated orally with a novel galacto-oligosaccharide. Human and Experimental Toxicology 33:814-821. | effects of paternal exposure cannot be separated from maternal ones |
| 19 | Läinen, J. K., P. Oskoei, M. Janhunen, H. Koskinen, R. Kortet, and H. Huuskonen. 2018. Sperm pre-fertilization thermal environment shapes offspring phenotype and performance. Journal of Experimental Biology 221. | exposure of gametes, not fathers |
| 20 | Lent, E. M., L. C. B. Crouse, A. M. Jackovitz, E. E. Carroll, and M. S. Johnson. 2016. An extended one-generation reproductive toxicity test of 1,2,4-Triazol-5-one (NTO) in rats. Journal of Toxicology and Environmental Health - Part A: Current Issues 79:1159-1178. | effects of paternal exposure cannot be separated from maternal ones |
| 21 | Li, Y., X. Lei, Z. Yin, W. Guo, S. Wu, and X. Yang. 2018. Transgenerational effects of paternal dietary Astragalus polysaccharides on spleen immunity of broilers. International Journal of Biological Macromolecules 115:90-97. | effects of paternal exposure cannot be separated from maternal ones |
| 22 | Lu, S. Y., J. W. Liao, M. L. Kuo, S. C. Wang, J. S. Hwang, and T. H. Ueng. 2004. Endocrine-disrupting activity in carbendazim-induced reproductive and developmental toxiclty in rats. Journal of Toxicology and Environmental Health - Part A 67:1501-1515. | effects of paternal exposure cannot be separated from maternal ones |
| 23 | Mao, Q. Z., W. Wu, Z. F. Liao, J. J. Li, D. S. Jia, X. F. Zhang, Q. Chen, H. Y. Chen, J. Wei, and T. Y. Wei. Viral pathogens hitchhike with insect sperm for paternal transmission. Nature Communications 10. | viral infection of the males was not induced experimentally |
| 24 | Mazer, S. J., and C. T. Schick. 1991. Constancy of population parameters for life-history and floral traits in Raphanus sativus L. II. Effects of planting density on phenotype and heritability estimates. Evolution 45:1888-1907. | males (polen donors) were grown under uniform conditions |
| 25 | Meems, L. M. G., H. Mahmud, H. Buikema, J. Tost, S. Michel, J. Takens, R. N. Verkaik-Schakel, I. Vreeswijk-Baudoin, I. V. Mateo-Leach, P. van Der Harst, T. Plösch, and R. A. de Boer. 2016. Parental vitamin d deficiency during pregnancy is associated with increased blood pressure in offspring via panx1 hypermethylation. American Journal of Physiology - Heart and Circulatory Physiology 311:H1459-H1469. | effects of paternal exposure cannot be separated from maternal ones |
| 26 | Morris, D. F., T. A. Marks, and G. M. Mesfin. 1987. Effects of losulazine on rat reproduction and development. Toxicological Sciences 9:573-587. | no data on offspring performance following paternal exposure |
| 27 | Neeper-Bradley, T. L., and B. Ballantyne. 2000. Two-generation reproduction study by dosing with glutaraldehyde in the drinking water of CD rats. Journal of Toxicology and Environmental Health - Part A 61:107-129. | effects of paternal exposure cannot be separated from maternal ones |
| 28 | O'Connor, J. C., S. M. Munley, T. L. Serex, and R. C. Buck. 2014. Evaluation of the reproductive and developmental toxicity of 6:2 fluorotelomer alcohol in rats. Toxicology 317:6-16. | effects of paternal exposure cannot be separated from maternal ones |
| 29 | Omkar, J. Sahu, and G. Kumar. 2010. Effect of prey quantity on reproductive and developmental attributes of a ladybird beetle, Anegleis cardoni. International Journal of Tropical Insect Science 30:48-56. | effects of paternal exposure cannot be separated from maternal ones |
| 30 | Parer, I., W. R. Sobey, D. Conolly, and R. Morton. 1995. Sire transmission of acquired-resistance to myxomatosis. Australian Journal of Zoology 43:459-465. | no proper control group |
| 31 | Paul, S. C., M. Stevens, J. K. Pell, M. A. Birkett, and J. D. Blount. 2018. Parental phenotype not predator cues influence egg warning coloration and defence levels. Animal Behaviour 140:177-186. | paternal phenotype was not determined experimentally |
| 32 | Penesova, A., J. C. Bunt, C. Bogardus, and J. Krakoff. 2010. Effect of paternal diabetes on pre-diabetic phenotypes in adult offspring. Diabetes Care 33:1823-1828. | study looks at the subset of offspring only (nondiabetic offspring) |
| 33 | Poulletier de Gannes, F., B. Billaudel, E. Haro, M. Taxile, L. Le Montagner, A. Hurtier, S. Ait Aissa, H. Masuda, Y. Percherancier, G. Ruffié, P. Dufour, B. Veyret, and I. Lagroye. 2013. Rat fertility and embryo fetal development: Influence of exposure to the Wi-Fi signal. Reproductive Toxicology 36:1-5. | effects of paternal exposure cannot be separated from maternal ones |
| 34 | Przybycien-Szymanska, M. M., Y. S. Rao, S. A. Prins, and T. R. Pak. 2014. Parental binge alcohol abuse alters f1 generation hypothalamic gene expression in the absence of direct fetal alcohol exposure. PLoS ONE 9. | effects of paternal exposure cannot be separated from maternal ones |
| 35 | Ripley, J. L., and C. M. Foran. 2009. Direct evidence for embryonic uptake of paternally-derived nutrients in two pipefishes (Syngnathidae: Syngnathus spp.). Journal of Comparative Physiology B: Biochemical, Systemic, and Environmental Physiology 179:325-333. | male exposure occurred after fertilization |
| 36 | Rodgers, A. B., C. P. Morgan, N. A. Leu, and T. L. Bale. 2015. Transgenerational epigenetic programming via sperm microRNA recapitulates effects of paternal stress. Proceedings of the National Academy of Sciences of the United States of America 112:13699-13704. | the study did not manipulate paternal phenotype |
| 37 | Shuey, D. L., D. G. Stump, R. D. Carliss, and R. J. Gerson. 2008. Effects of the opioid analgesic oxymorphone hydrochloride on reproductive function in male and female rats. Birth Defects Research Part B - Developmental and Reproductive Toxicology 83:12-18. | effects of paternal exposure cannot be separated from maternal ones |
| 38 | Smallegange, I. M. 2011. Effects of paternal phenotype and environmental variability on age and size at maturity in a male dimorphic mite. Naturwissenschaften 98:339-346. | expression of the two alternative reproductive phenotypes is partly genetically determined |
| 39 | Soares, J., A. M. Coimbra, M. A. Reis-Henriques, N. M. Monteiro, M. N. Vieira, J. M. A. Oliveira, P. Guedes-Dias, Fontaínhas-Fern, A. es, S. S. Parra, A. P. Carvalho, L. F. C. Castro, and M. M. Santos. 2009. Disruption of zebrafish (Danio rerio) embryonic development after full life-cycle parental exposure to low levels of ethinylestradiol. Aquatic Toxicology 95:330-338. | effects of paternal exposure cannot be separated from maternal ones |
| 40 | Sowers, A. D., K. M. Gaworecki, M. A. Mills, A. P. Roberts, and S. J. Klaine. 2009. Developmental effects of a municipal wastewater effluent on two generations of the fathead minnow, Pimephales promelas. Aquatic Toxicology 95:173-181. | effects of paternal exposure cannot be separated from maternal ones |
| 41 | Stein, L. R., S. A. Bukhari, and A. M. Bell. 2018. Personal and transgenerational cues are nonadditive at the phenotypic and molecular level. Nature Ecology and Evolution 2:1306-1311. | male exposure to predator occurred after fertilization |
| 42 | Szutorisz, H., G. Egervári, J. Sperry, J. M. Carter, and Y. L. Hurd. 2016. Cross-generational THC exposure alters the developmental sensitivity of ventral and dorsal striatal gene expression in male and female offspring. Neurotoxicology and Teratology 58:107-114. | effects of paternal exposure cannot be separated from maternal ones |
| 43 | Szutorisz, H., J. A. DiNieri, E. Sweet, G. Egervari, M. Michaelides, J. M. Carter, Y. Ren, M. L. Miller, R. D. Blitzer, and Y. L. Hurd. 2014. Parental THC exposure leads to compulsive heroin-seeking and altered striatal synaptic plasticity in the subsequent generation. Neuropsychopharmacology 39:1315-1323. | effects of paternal exposure cannot be separated from maternal ones |
| 44 | Takakura, K. I. Variation in egg size within and among generations of the bean weevil, Bruchidius dorsalis (Coleoptera, Bruchidae): Effects of host plant quality and paternal nutritional investment. Annals of the Entomological Society of America 97:346-352. | the only offspring trait measured was egg size, which is a trait under maternal control |
| 45 | Tanaka, T., O. Takahashi, A. Inomata, A. Ogata, and D. Nakae. 2012. Reproductive and Neurobehavioral Effects of Brilliant Blue FCF in Mice. Birth Defects Research Part B - Developmental and Reproductive Toxicology 95:395-409. | effects of paternal exposure cannot be separated from maternal ones |
| 46 | Tracey, R., M. Manikkam, C. Guerrero-Bosagna, and M. K. Skinner. 2013. Hydrocarbons (jet fuel JP-8) induce epigenetic transgenerational inheritance of obesity, reproductive disease and sperm epimutations. Reproductive Toxicology 36:104-116. | effects of male in utero exposure cannot be separated from maternal ones |
| 47 | Tyl, R. W., C. B. Myers, M. C. Marr, C. S. Sloan, N. P. Castillo, M. M. Veselica, J. C. Seely, S. S. Dimond, J. P. Van miller, R. S. Shiotsuka, G. D. Stropp, J. M. Waechter, and S. G. Hentges. 2008. Two-generation reproductive toxicity evaluation of dietary 17β-Estradiol (E2; CAS No. 50-28-2) in CD-1 (Swiss) mice. Toxicological Sciences 102:392-412. | effects of paternal exposure cannot be separated from maternal ones |
| 48 | Tyl, R. W., C. B. Myers, M. C. Marr, P. A. Fail, J. C. Seely, B. Elswick, A. James, and F. Welsch. 2003. Two-generation reproductive toxicity study in inhaled tertiary amyl methyl ether (TAME) vapor in CD® rats. Journal of Applied Toxicology 23:397-410. | effects of paternal exposure cannot be separated from maternal ones |
| 49 | Tyl, R. W., C. B. Myers, M. C. Marr, P. A. Fail, J. C. Seely, D. R. Brine, R. A. Barter, and J. H. Butala. 2004. Reproductive toxicity evaluation of dietary butyl benzyl phthalate (BBP) in rats. Reproductive Toxicology 18:241-264. | effects of paternal exposure cannot be separated from maternal ones |
| 50 | Velasco-Santamaría, Y. M., R. D. y, and K. A. Sloman. 2011. Endosulfan affects health variables in adult zebrafish (Danio rerio) and induces alterations in larvae development. Comparative Biochemistry and Physiology - C Toxicology and Pharmacology 153:372-380. | effects of paternal exposure cannot be separated from maternal ones |
| 51 | Vijendravarma, R. K., S. Narasimha, and T. J. Kawecki. 2010. Effects of parental larval diet on egg size and offspring traits in Drosophila. Biology Letters 6:238-241. | effects of paternal exposure cannot be separated from maternal ones |
| 52 | Viktil, K. K., Engel, A. , and K. Furu. 2009. Use of antirheumatic drugs in mothers and fathers before and during pregnancy - A population-based cohort study. Pharmacoepidemiology and Drug Safety 18:737-742. | no information on offspring performance |
| 53 | Vincent, C. M., and D. T. Gwynne. 2014. Sex-biased immunity is driven by relative differences in reproductive investment. Proceedings of the Royal Society B: Biological Sciences 281. | no assessment of the offspring |
| 54 | Xu, M., Y. Ma, L. Xu, Y. Xu, and Y. Li. 2012. Multigenerations Assessment of Dietary Nucleotides Consumption in Weaned Rats. Birth Defects Research Part B - Developmental and Reproductive Toxicology 95:460-466. | effects of paternal exposure cannot be separated from maternal ones |
| 45 | Yang, D., X. T. Lai, L. Sun, and F. S. Xue. 2007. Parental effects: Physiological age, mating pattern, and diapause duration on diapause incidence of progeny in the cabbage beetle, Colaphellus bowringi Baly (Coleoptera: Chrysomelidae). Journal of Insect Physiology 53:900-908. | effects of paternal exposure cannot be separated from maternal ones |
| 56 | Zhang, M. X., W. J. Xu, G. He, D. Zhang, X. L. Zhao, J. B. Dai, J. J. Wu, Y. Cao, Z. X. Wang, L. Y. Wang, and Z. D. Qiao. Maternal nicotine exposure has severe cross-generational effects on offspring behavior. Behavioural Brain Research 348:263-266. | effects of paternal exposure cannot be separated from maternal ones |
| 57 | Zhang, X. G., H. Zhang, L. Lin, Y. Q. Yang, T. T. Deng, Q. Liu, X. L. Liang, M. Q. Wang, and d. Peng. 2014. Genes underlying positive influence of prenatal environmental enrichment and negative influence of prenatal earthquake simulation and corrective influence of Chinese herbal medicine on rat offspring: Irf7 and Ninj2. African journal of traditional, complementary, and alternative medicines : AJTCAM / African Networks on Ethnomedicines 11:367-376. | no data of offspring performance following paternal in utero exposure |
| 58 | Zhou, R., G. Lu, Z. Yan, R. Jiang, J. Shen, and X. Bao. 2019. Parental transfer of ethylhexyl methoxy cinnamate and induced biochemical responses in zebraﬁsh. Aquatic Toxicology:24-32. | effects of paternal exposure cannot be separated from maternal ones |
| 59 | Burov, N. E., E. V. Arzamastsev, L. I. Kornienko, and L. A. Kudimova. 2002. Influence of xenon on reproductive function. Anesteziologiya i Reanimatologiya:71-72. | full text not in English |
| 60 | Chang, F., Z. Meng, and H. Wang. 2006. Dominant lethal effect of sulfur dioxide derrivatives on mice. Chinese Journal of Applied and Environmental Biology **12**:360-362 | full text not in English |
| 61 | Duan, M. N., Y. J. Liu, X. Bai, X. Gao, X. X. Zhang, and D. Q. Xiong. 2018. Exposure of adult sea urchins to sunken heavy fuel oil affects the reproductive status and the development of their offspring. Zhongguo Huanjing Kexue/China Environmental Science **38**:4720-4729. | full text not in English |
| 62 | Li, J. N., Y. Song, J. B. Li, Z. G. Liu, X. Y. Chen, Q. J. Zhang, and X. Q. Xiao. 2013. Effects of streptozocin-induced hyperglycemia in male rats on growth, development and metabolism of offspring. Chinese Journal of Biologicals **26**:505-508+511 | full text not in English |
| 63 | Okada, F., F. Sagami, P. Tirone, A. Morisetti, S. Bussi, and J. K. Baguley. 1999. Reproductive and developmental toxicity study of gadobenate dimeglumine formulation (E7155) (1) - Fertility study in male rats by intravenous administration. Journal of Toxicological Sciences **24**:61-69. | full text not in English |
